# Oesophageal squamous cell neoplasia in head and neck cancer patients: upregulation of COX-2 during carcinogenesis

**DOI:** 10.1038/sj.bjc.6600865

**Published:** 2003-04-15

**Authors:** K Maaser, P Däubler, B Barthel, B Heine, B von Lampe, H Stein, B Hoffmeister, H Scherer, H Scherübl

**Affiliations:** 1 Medical Clinic I, Gastroenterology/Infectious Diseases/Rheumatology, University Hospital Benjamin Franklin, Free University of Berlin, 12200 Berlin, Germany; 2 Institute of Pathology, University Hospital Benjamin Franklin, Free University of Berlin, 12200 Berlin, Germany; 3 Department of Maxillofacial Plastic Surgery, University Hospital Benjamin Franklin, Free University of Berlin, 12200 Berlin, Germany; 4 ENT Department, University Hospital Benjamin Franklin, Free University of Berlin, 12200 Berlin, Germany

**Keywords:** carcinogenesis, COX-2, oesophageal squamous cell carcinoma, head and neck cancer

## Abstract

Patients with (previous) head and neck cancer (HNC) are at high risk for developing second squamous cell cancer of the oesophagus. The role of cyclooxygenase-2 (COX-2) in oesophageal squamous carcinogenesis has not yet been investigated in this high-risk group. Therefore, this study examined COX-2 mRNA and protein expression in oesophageal biopsies and resected tissues of 44 HNC patients. The evaluation covered 55 oesophageal tissue samples (18 invasive oesophageal squamous cell cancers, four high- and eight low-grade dysplasias, 25 normal squamous epithelia) from the 44 patients. mRNA levels of COX-2 were measured by real-time PCR using a LightCycler. COX-2 protein expression was studied immunohistochemically and graded by a staining score. COX-2 mRNA was detected in all samples, and its levels correlated positively with the immunohistochemical staining score (*P*<0.05). COX-2 expression was upregulated during oesophageal squamous carcinogenesis in HNC patients, that is COX-2 expression increased significantly from normal oesophageal squamous epithelium to low- and high-grade dysplasia and finally to invasive squamous cell cancer (*P*<0.001). Our findings suggest that COX-2 upregulation contributes to oesophageal squamous carcinogenesis in HNC patients. Prospective studies are needed to evaluate the chemopreventive potential of COX-2 inhibitors in this high-risk group.

The overall prognosis of oesophageal cancer is poor due to advanced disease at diagnosis (Gabbert *et al*, 2000). Only the small group of patients who are diagnosed at an early stage (T_is_N_0_M_0_, T_1a_N_0_M_0_) have very favourable prospects (Makuuchi *et al*, 1996; Ell *et al*, 2000). Groups at high risk for oesophageal cancer should thus be identified, monitored, and submitted to effective chemoprevention. The development of oesophageal squamous cell carcinoma (ESCC) is thought to be a multistep process involving oesophagitis, atrophy, mild to severe dysplasia, carcinoma *in situ*, and, finally, invasive ESCC. Genetic changes associated with the development of ESCC include mutations of the p53 gene, disruption of cell-cycle control, activation of oncogenes, and inactivation of several tumour suppressor genes (Mandard *et al*, 2000). Oesophageal carcinogenesis and tumour progression require more detailed elucidation, particularly in high-risk groups. Patients with (previous) head and neck cancer (HNC) are a clinically important high-risk group with an 11.8–16.6% risk of ESCC or intraepithelial neoplasia (Makuuchi *et al*, 1996; Tincani *et al*, 2000; Scherübl *et al*, 2002b). Thus, effective chemoprevention of oesophageal neoplasia is most desirable in these patients.

The cyclooxygenase (COX) enzymes catalyse the formation of eicosanoids including prostaglandins from arachidonic acid. There are two distinct isoforms of COX. COX-1 is constitutively expressed in most mammalian tissues. In contrast, COX-2 mRNA and protein are either undetectable or expressed only at low levels in most tissues (Smith *et al*, 1996). COX-2 is an immediate-early response gene induced by inflammation, growth factors, tumour promoters, oncogenes, and carcinogens (Kutchera *et al*, 1996; Smith *et al*, 1996). Recent studies have highlighted the relevance of COX-2 expression in gastrointestinal carcinogenesis. COX-2 is overexpressed in transformed cells and cancers of both the pharynx and gastrointestinal tract, including squamous carcinoma of the head and neck (Chan *et al*, 1999), colorectal carcinoma (Kutchera *et al*, 1996), gastric carcinoma (Ristimaki *et al*, 1997), Barrett's oesophagus and Barrett's adenocarcinomas (Wilson *et al*, 1998; Zimmermann *et al*, 1999; Morris *et al*, 2001), as well as ESCC (Ratnasinghe *et al*, 1999; Zimmermann *et al*, 1999; Shamma *et al*, 2000). Overexpression of COX-2 is associated with increased proliferation, reduced apoptosis (Tsujii and Dubois, 1995), stimulation of angiogenesis (Jones *et al*, 1999), and invasive or metastatic potential (Tsujii *et al*, 1997). Since all these factors contribute to oesophageal carcinogenesis and cancer progression, COX-2 appears to be a promising target protein for innovative chemoprevention strategies (Kawamori *et al*, 1998; Harris *et al*, 2000; Steinbach *et al*, 2000; Buttar *et al*, 2002; Thun *et al*, 2002; Zweifel *et al*, 2002).

Here we report on COX-2 upregulation during oesophageal squamous carcinogenesis in HNC patients. We performed real-time PCR with a LightCycler to quantify COX-2 mRNA expression and used immunohistochemistry to study cellular COX-2 protein expression.

## MATERIAL AND METHODS

### Patients and tissue samples

Normal, dysplastic, or malignant oesophageal tissues were collected from 44 HNC patients and a control group of nine other patients (without HNC or oesophageal disease). The human tumour material was used according to the standards set by the Ethics Committee of the University Hospital Benjamin Franklin, Free University of Berlin. The group of HNC patients comprised 33 men and 11 women with an average age of 60.0 years (range 45–89 years).

The histological diagnosis (normal, dysplastic, or malignant oesophageal tissue) was based on an independent review of the cases by two to three observers. If dysplasia was found, the microsections were evaluated by three different pathologists. All observers agreed on the diagnosis of cancer or dysplasia in the patients described here. Two out of three was considered a consensus for specimens without unanimous dysplasia grading (Scherübl *et al*, 2002b). Low- and high-grade dysplasia (synonym: intraepithelial neoplasia) was defined as unequivocal neoplastic transformation according to previously published criteria (Lewin and Appelman, 1995).

Eighteen of the 44 HNC patients had ESCC. Dysplastic oesophageal lesions (one low-grade dysplasia (LGD), two high-grade dysplasias (HGD)) were available for evaluation from three of the 18 ESCC patients and normal oesophageal squamous epithelia from eight of them. In addition, nine oesophageal dysplasias (seven LGD, two HGD) and 17 normal oesophageal squamous epithelia could be studied from the 26 HNC patients without ESCC. Most tissue samples were obtained during an endoscopic screening study for oesophageal neoplasia in HNC patients; the protocol included systematic biopsies along the squamous oesophagus (Scherübl *et al*, 2002a,2002b). Patients were oesophagoscoped using high-resolution videoendoscopes (Olympus GIF-140, Hamburg, Germany). Biopsy specimens were obtained with the 7.5 mm open span biopsy forceps (Endoflex K02 22 V-A).

### RNA purification and cDNA construction

Total RNA was isolated from oesophageal squamous tissue biopsies using the RNeasy Kit (Qiagen, Santa Clarita, CA, USA) according to the manufacturer's instructions. Briefly, the biopsies were lysed in 350 *μ*l of lysis buffer for 3 × 10 s using an ultrasonic disintegrator (Sonoplus HD 70, Bandelin Electronic, Berlin, Germany) and homogenised using the QIAshredder Kit (Qiagen, Santa Clarita, CA, USA). After centrifugation, total RNA was purified from the supernatant by affinity chromatography using the RNeasy columns. Aliquots of 1.5 *μ*g of total RNA were further purified by DNAse I (Gibco, Rockville, MD, USA) digestion and reverse transcribed into cDNA using oligo-dT-primers and the SuperScript Preamplification Kit (Gibco, Rockville, MD, USA).

### LightCycler PCR

Each 10 *μ*l reaction volume contained 1 × FastStart DNA Master Hybridization Probes Mix (Roche Diagnostics, Mannheim, Germany), 2 mM of MgCl_2_, 0.5 *μ*M of each primer, 0.2 *μ*M of each hybridisation probe (fluorescein and LC-Red640), and 1 *μ*l of cDNA. The housekeeping gene porphobilinogen deaminase (PBGD) was selected for reference because of its relatively low expression (comparable to that of COX-2) and lack of pseudogenes. The amplified fragments measured 198 bp (COX-2) and 282 bp (PBGD) (Table 1

**Table 1:** Sequences of primers and hybridisation probes used

**Gene**	**Primer (5′3′)**	**Location**	**Gene Bank**
COX-2			
Sense	CCTCCTgTgCCTgATgATTgC	549–569	NM_000963
Antisense	TggCCCTCgCTTATgATCTg	727–746	
Fluorescein	ATCCCCAgggCTCAAACATgATgT-*FL*	661–684	
LC 640	*LC640*-CATTCTTTgCCCAgCACTTCACgC-*p*	688–711	
			
PBGD			
Sense	AgAgTgATTCgCgTgggTACC	82–102	NM_000190
Antisense	GgCTCCgATggTgAAgCC	346–363	
Fluorescein	AgTggACCTggTTgTTCACTCCTTgAA-*FL*	294–320	
LC 640	*LC640*-ACCTgCCCACTgTgCTTCCTCCT-*p*	323–345	

). Real-time PCR was performed in a LightCycler (Roche Diagnostics, Mannheim, Germany) under the following conditions: initial heating to 95°C for 10 min, then 40 cycles at 95°C for 0 s, 64°C for 12 s, and 72°C for 12 s. The COX-2 and PBGD expressions were quantified using external standards. These standards were set up by PCR using primers that amplify COX-2 and PBGD fragments with ends about 40–150 bases longer (primer sequences and reaction conditions not shown). These fragments were reamplified and purified several times. The concentration of the standards was adjusted to 10 ng *μ*l^−1^, and serial dilutions were carried out in 20 ng *μ*l^−1^ polyA (Pharmacia, Uppsala, Sweden). Separate standard curves were included for COX-2 and PBGD in each PCR run. Copy numbers of each sample were calculated from the standard curve. The ratio of COX-2 to PBGD copy numbers was calculated to compare COX-2 expression between specimens.

### Immunohistochemistry

Microsections (2–3 *μ*m) of paraffin-embedded primary tumours were deparaffinised and rehydrated in a decreasing alcohol series. Immunohistochemistry was performed using a robotic system (Chemo-mate, DAKO, Heidelberg, Germany). Sections were incubated with the monoclonal anti-COX-2 antibody (5 *μ*g ml^−1^, Cayman Chemical Company, Ann Arbor, MI, USA) for 30 min at room temperature (Sinicrope *et al*, 1999). After washing, samples were incubated with anti-mouse IgG (1 : 20 dilution) for 30 min at room temperature, and staining was detected by the ‘fast-red system’ (DAKO, Heidelberg, Germany). Samples were slightly counterstained in Mayer's haematoxylin.

Tissue staining was independently scored by two of the authors (P. Däubler and B. Heine) with an interobserver discrepancy of less than 10%. In case of discrepancy, consensus interpretation was reached with a third author (H. Stein). The increase in positive tumour cells was graded as follows: 0=none, 1=weak, 2=moderate, 3=strong. A score of 0–12 was calculated as the product of the increase in staining intensity and the frequency of stained cancer cells (0=0%, 1=1–25%, 2=26–50%, 3=51–75%, 4=76–100%). Since some tumours displayed inhomogeneous staining patterns, each tumour component was scored independently, and the results were totalled. All tissues contained positive nonepithelial cells such as lymphocytes which served as a positive internal control.

### Statistical analysis

Statistical analysis was performed using the PRISM Statistic Package 2.0 (Graph-Pad Software Inc., San Diego, CA, USA). Groups were compared using the Kruskal–Wallis test (nonparametric analysis of variance) and were considered different at *P*<0.05. In these cases, *P* values for comparing groups were calculated using Dunn's multiple comparisons as a nonparametric post-test. Correlations were computed by the Spearman rank test. The correlation between COX-2 protein and mRNA expression was analysed using the nonparametric two-tailed Spearman test.

## RESULTS

### Quantitative analysis of COX-2 mRNA expression

**Figure 1:**
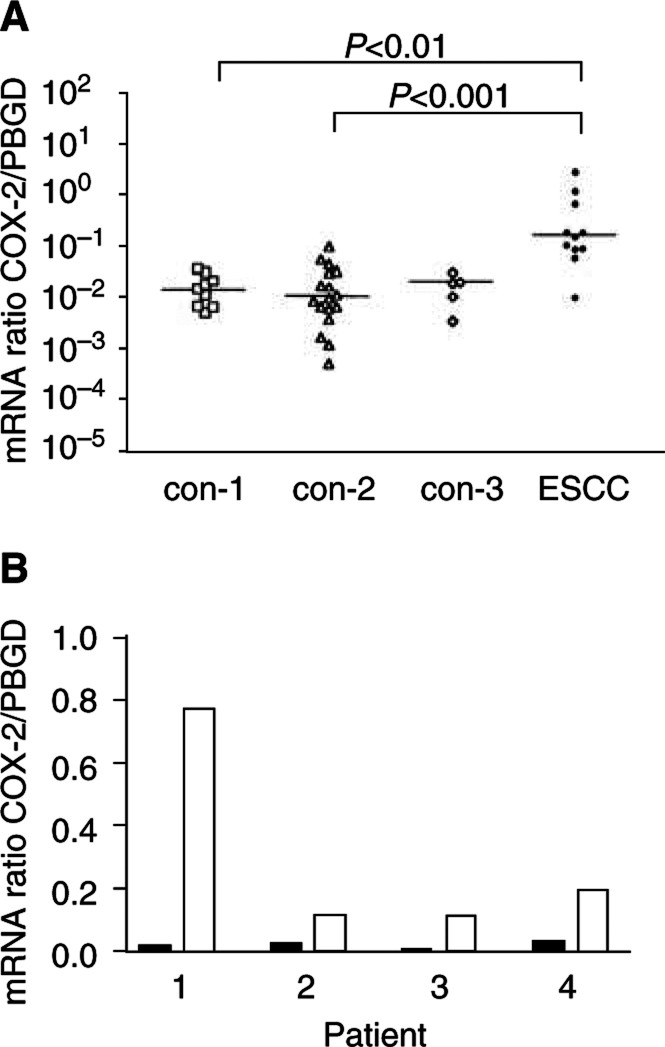
COX-2 mRNA expression in normal and neoplastic squamous tissues of the oesophagus of HNC patients. COX-2 mRNA levels were normalised to PBGD. (**A**) Comparison between normal and neoplastic oesophageal tissues: con-1.=normal oesophageal squamous epithelium from control patients without HNC or oesophageal disease; con-2=normal oesophageal squamous epithelium from HNC patients without oesophageal neoplasia; con-3=normal oesophageal squamous epithelium from patients with both HNC and ESCC. (**B**) COX-2 levels normalised to PBGD in ESCC tissues (white columns) and the corresponding normal oesophageal squamous epithelium (black columns) of four individual HNC patients. HNC=head and neck cancer.

COX-2 mRNA expression was examined using real-time PCR. Both normal oesophageal squamous epithelium and ESCC were investigated for COX-2 expression. Normal oesophageal squamous epithelium showed consistently low COX-2 mRNA expression, and no difference was found between control patients without HNC or oesophageal disease (con-1 in Figure 1A

), HNC patients without oesophageal disease (con-2 in Figure 1A), and HNC patients with ESCC (con-3 in Figure 1A). In contrast, COX-2 mRNA expression was significantly increased in ESCC (of HNC patients). A direct comparison between ESCC tissue and normal oesophageal epithelium in the same patient revealed marked overexpression of COX-2 in the cancer tissue (Figure 1B). These findings point to an upregulation of COX-2 during carcinogenesis.

Dysplasias can be very small lesions that are often closely associated with either adjacent normal epithelium or carcinoma tissue. Dysplastic lesions were studied by immunohistochemistry alone to obtain clear evidence of their COX-2 (over)expression and to exclude possible contamination by nondysplastic tissue.

### Immunohistochemical analysis of COX-2 expression

**Figure 2:**
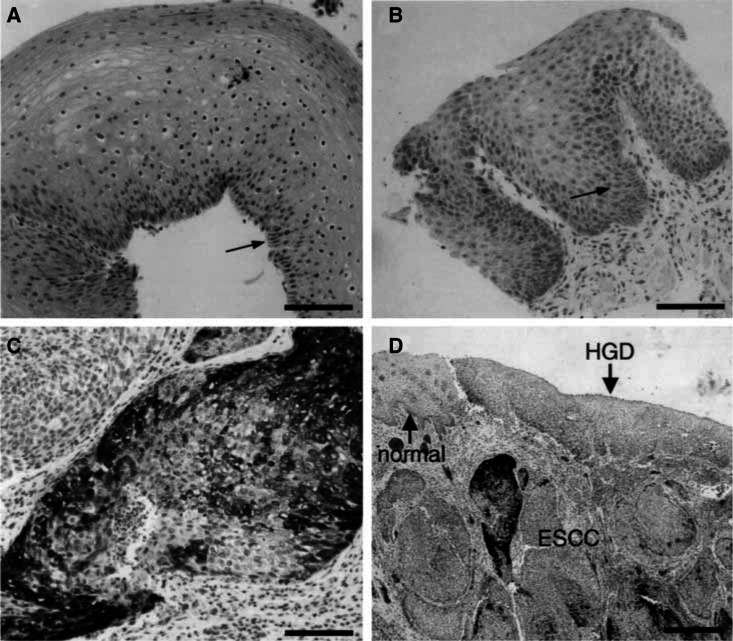
COX-2 immunoreactivity in normal and neoplastic oesophageal squamous tissues of HNC patients. (**A**) Normal oesophageal squamous epithelium exhibits COX-2-specific staining only in cells of the basal layer (arrow); the staining score is 1. (**B**) Squamous cells of LGD demonstrate moderate COX-2-specific staining; the staining score is 4. (**C**) Poorly differentiated ESCC shows heterogeneous COX-2 staining; the staining score is 4. (A–C) Bar=100 *μ*M. (**D**) Resected oesophageal tissue showing COX-2 expression that increases from normal squamous mucosa (normal) to HGD and to carcinoma (ESCC). Bar=500 *μ*M.

**Figure 3:**
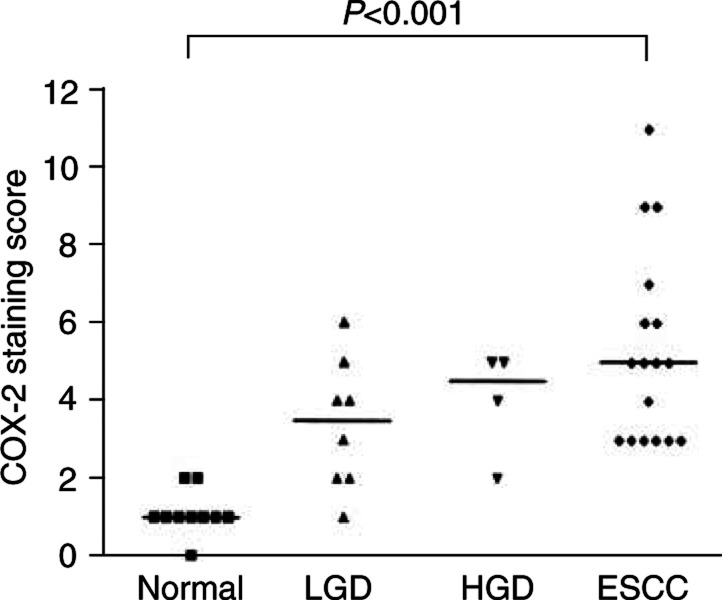
COX-2 immunoreactivity score of normal and neoplastic oesophageal squamous tissues in HNC patients. The immunoreactivity score is the product of the percentage of positive cells and the staining intensity. COX-2 expression is significantly higher in ESCC than in normal epithelium. LGD=low-grade dysplasia; HGD=high-grade dysplasia of the squamous oesophagus.

COX-2 was mainly expressed in the basal layer of normal squamous epithelium (Figure 2A

, arrow). COX-2-positive cells comprised about 20%. Weak COX-2-specific staining was generally detected in the cytoplasm. The median staining score was 1 (Figure 3

). COX-2 showed higher expression in dysplastic than in normal epithelial cells. It was detected in the cytoplasm of most dysplastic cells, generally with a homogeneously weak to moderate intensity (Figure 2B). Low-grade dysplasia and HGD showed no marked differences in COX-2-specific staining. The median staining score was 3.5 for LGD and 4.5 for HGD (Figure 3). COX-2 expression was significantly stronger in ESCC than in normal squamous epithelium (*P*<0.001). Here the median staining score was 5. In contrast to dysplastic cells, ESCC cells often displayed heterogeneous COX-2 expression pattern, and the staining intensity could vary from weak to strong within the same tumour (Figure 2C). COX-2 mRNA levels correlated significantly with the immunoreactive COX-2 protein expression of the same tumour (*r*=0.64, *P*<0.05), indicating that COX-2 expression is regulated at the mRNA level and that COX-2 mRNA analysis by real-time PCR is an adequate tool for determining COX-2 expression.

## DISCUSSION

The incidence of oesophageal cancer shows striking geographic variation. In high-risk areas like Linxian, China, mass surveillance programmes for oesophageal cancer have been implemented to reduce the high mortality rate of 161 out of 100 000 (Wu *et al*, 1982). In Western countries, the incidence of ESCC is generally below 6 out of 100 000 (Fleischer and Haddad, 1999; Gabbert *et al*, 2000). The rarity of this malignancy does not justify implementing a costly endoscopic surveillance programme for the general population in low-risk areas. Even in those areas, however, surveillance and chemoprevention may be warranted in high-risk groups. High-risk groups for ESCC include male subjects with heavy drinking or smoking habits, patients with (previous) HNC, patients with corrosive oesophagitis or carriers of the gene for autosomal dominant transmission of tylosis (Makuuchi *et al*, 1996; Scherübl *et al*, 2002b). The overall prognosis of oesophageal cancer is very poor due to advanced disease at diagnosis and aggressive tumour biology. Surveillance and chemoprevention must therefore be considered for high-risk groups such as HNC patients (Ratnasinghe *et al*, 1999; Zimmermann *et al*, 1999; Shamma *et al*, 2000). Several epidemiological studies indicate that the use of aspirin or other nonsteroidal anti-inflammatory drugs may reduce the risk of death from both colorectal and oesophageal cancer (Thun *et al*, 1993; Funkhouser and Sharp, 1995; Thun *et al*, 2002). The relevance of COX-2 in human carcinogenesis has been highlighted by many recent studies (Fosslien, 2000; Shamma *et al*, 2000; Morris *et al*, 2001; Cao and Prescott, 2002). Blockade or even reversal of the multistep process of carcinogenesis should be targeted in high-risk groups. In our study on HNC patients, a well-known high-risk group for ESCC, we observed a marked COX-2 overexpression in both oesophageal squamous cell dysplasias and ESCC. Normal oesophageal squamous epithelium (even in HNC patients) showed very low COX-2 expression. Starting from that very low level, COX-2 expression increased stepwise in the sequence of normal epithelium, oesophageal squamous cell dysplasia, and finally ESCC (Shamma *et al*, 2000). Interestingly, COX-2 expression in ESCC of HNC patients shows several similarities to that in ESCC patients without HNC: low homogeneous COX-2 expression in normal epithelium, increasing expression during carcinogenesis, homogeneous COX-2 distribution in all dysplasias, and intra- and intertumoural heterogeneity of COX-2 expression in carcinoma tissues (Zimmermann *et al*, 1999; Ratnasinghe *et al*, 1999; Shamma *et al*, 2000).

In this study, COX-2 expression was quantified by real-time PCR and immunohistochemistry. COX-2 mRNA levels correlated positively with the immunohistological COX-2 staining score, indicating that COX-2 expression is regulated on a transcriptional level in oesophageal neoplasias. This positive correlation implies that quantitative COX-2 mRNA measurement in biopsies is an adequate method for determining COX-2 expression in both normal and neoplastic oesophageal squamous epithelium. Interestingly, other COX-2-positive cell types such as immune or stroma cells must have comprised only a very small fraction, since they did not affect the results.

The mechanisms by which elevated COX-2 expression interferes with carcinogenesis and tumour progression are the object of ongoing research, but several cellular processes have already been pointed out. Selective COX-2 inhibition has been shown to induce apoptosis and reduce proliferation of oesophageal cancer cells. These effects correlate with the inhibition of prostaglandin synthesis (Zimmermann *et al*, 1999). Moreover, COX-2 can influence apoptosis by decreasing the production of the apoptosis mediator ceramide or by upregulating the antiapoptotic protein Bcl-2 (Tsujii and Dubois, 1995; Chan *et al*, 1998). Other possible antineoplastic actions of COX-2 blockers include cell cycle control (Yu *et al*, 1995), regulation of cell adhesion (Tsujii and Dubois, 1995), immune function (Tsujii *et al*, 1997), and inhibition of angiogenesis and neovascularisation (Jones *et al*, 1999). Thus nonsteroidal anti-inflammatory drugs, especially specific COX-2 inhibitors, are being discussed as anticancer agents, and numerous experimental, clinical, and epidemiological studies have demonstrated their antineoplastic effects (reviewed in Thun *et al*, 2002).

In summary, our study adds to the growing evidence that COX-2 upregulation plays an important role in oesophageal carcinogenesis in general and for the first time in a high-risk group of ESCC such as HNC patients. As aspirin, a nonselective COX inhibitor, reduces the risk of oesophageal cancer (Thun *et al*, 1993; Funkhouser and Sharp, 1995) and as specific COX-2 inhibitors suppress tumorigenesis both in experimental tumour models (Kawamori *et al*, 1998; Harris *et al*, 2000; Buttar *et al*, 2002; Thun *et al*, 2002; Zweifel *et al*, 2002) and in diseases such as human familial adenomatous polyposis (Steinbach *et al*, 2000), prospective clinical studies are needed to evaluate the chemopreventive potential of COX-2 inhibitors. Due to an ESCC risk of 10% and more, HNC patients should be enrolled in chemoprevention trials with COX-2 inhibitors. The benefit of effective chemoprevention will have to outweigh putative side effects of COX-2 inhibitors (Fosslien, 2000; Thun *et al*, 2002).
